# Bilateral obstructive uropathy and severe renal dysfunction associated with large prolapsed pedunculated submucosal leiomyoma of the uterus misdiagnosed as an intracervical fibroid: Report of a very rare case and a mini‑review of the literature 

**DOI:** 10.3892/mi.2024.150

**Published:** 2024-03-27

**Authors:** Efthymia Thanasa, Anna Thanasa, Vasiliki Grapsidi, Emmanouil Xydias, Evangelos Kamaretsos, Apostolos Ziogas, Ioannis Paraoulakis, Evagelia Simopoulou, Maria Mousia, Ioannis Thanasas

**Affiliations:** 1 Department of Health Sciences, Medical School, Aristotle University of Thessaloniki, 54124 Thessaloniki, Greece; 2 Department of Obstetrics and Gynecology, General Hospital of Trikala, 42100 Trikala, Greece; 3 Department of Obstetrics and Gynaecology, EmbryoClinic IVF, 55133 Thessaloniki, Greece; 4 Department of Medicine, University of Thessaly, School of Health Sciences, 41334 Larissa, Greece; 5 Department of Pathology, General Hospital of Trikala, 42100 Trikala, Greece

**Keywords:** pedunculated submucosal leiomyoma, intracervical leiomyoma, obstructive uropathy, renal dysfunction, ultrasound, computed tomography, magnetic resonance imaging, surgical treatment

## Abstract

Pedunculated submucosal leiomyomas of the uterus that prolapse into the vagina are common. In extremely rare cases, large pedunculated submucosal leiomyomas may lead to bilateral obstructive uropathy, causing severe renal dysfunction and potentially being misdiagnosed as intracervical leiomyoma. The present study describes the surgical treatment of a patient with a large prolapsed pedunculated submucosal uterine leiomyoma, which was misdiagnosed as an intracervical fibroid. The patient, of menopausal age, presented with uterine bleeding, anemia and severe renal dysfunction. Upon a physical examination, suspicion arose for a cervical leiomyoma, prompting the decision for imaging. Both transvaginal ultrasound and computed tomography, as well as magnetic resonance imaging confirmed the diagnosis of intracervical leiomyoma, accompanied by bilateral obstructive uropathy due to ureteral compression. The surgical management of the patient with laparotomy was decided. Intraoperatively, a large pedunculated submucosal uterine leiomyoma prolapsing into the vagina was identified. Total hysterectomy and bilateral salpingectomy-oophorectomy were performed. The immediate post-operative course was uneventful. At 6 months following surgery, the complete recovery of renal morphology and function was observed. The patient continues to undergo regular follow-up assessment to date. In the present study, a brief literature review is also provided, emphasizing the significant diagnostic and surgical challenges that may arise in the management of patients with large pedunculated submucosal uterine leiomyomas prolapsing into the vagina.

## Introduction

Uterine fibroids or leiomyomas are benign pelvic masses, representing the most common gynecological condition to date (1). There are three primary types of uterine leiomyomas: Submucosal, intramural and subserosal leiomyomas. Submucosal and subserosal leiomyomas may be attached to the uterus via a vascular pedicle, known as pedunculated leiomyomas (2). Depending on their location, pedunculated uterine leiomyomas may protrude both within the uterine cavity or extend outside it, into the vagina (as in the case described herein). Alternatively, they may prolapse outside the vagina or protrude into the peritoneal cavity, often posing a serious challenge for differential diagnosis (3).

The case described in the present study emphasizes the significant obstructive bilateral hydroureteronephrosis, accompanied by severe renal dysfunction resulting from ureteral compression by a large pedunculated submucosal uterine leiomyoma that prolapsed into the vagina. Simultaneously, it highlights the substantial challenges in differential diagnosis, particularly with large intracervical leiomyomas, and emphasizes the imperative need for early and effective surgical intervention to prevent permanent damage of the renal parenchyma.

## Case report

The present study describes the case of a 65-year-old menopausal patient, who presented to the Emergency Department of the General Hospital of Trikala (Trikala, Greece) with severe uterine bleeding persisting for 20 days, leading to marked anemia. The blood tests of the patient upon admission to the Department of Gynecology the General Hospital of Trikala are presented in detail in Table I. The patient had given birth to two children by vaginal delivery. From the personal medical history of the patient, it was found that she had no prior surgeries. Conditions, such as hypothyroidism secondary to non-toxic multinodular goiter, chronic atrial fibrillation, arterial hypertension and type II diabetes mellitus were reported. These conditions were effectively managed with medication. The patient reported no bowel disorders or weight loss. Sporadic episodes of frequent urination, without accompanying urinary tract infection, were mentioned by the patient. Additionally, there was no history of chronic renal disease or recurrent urinary tract infections in recent years.

**Figure 1:**
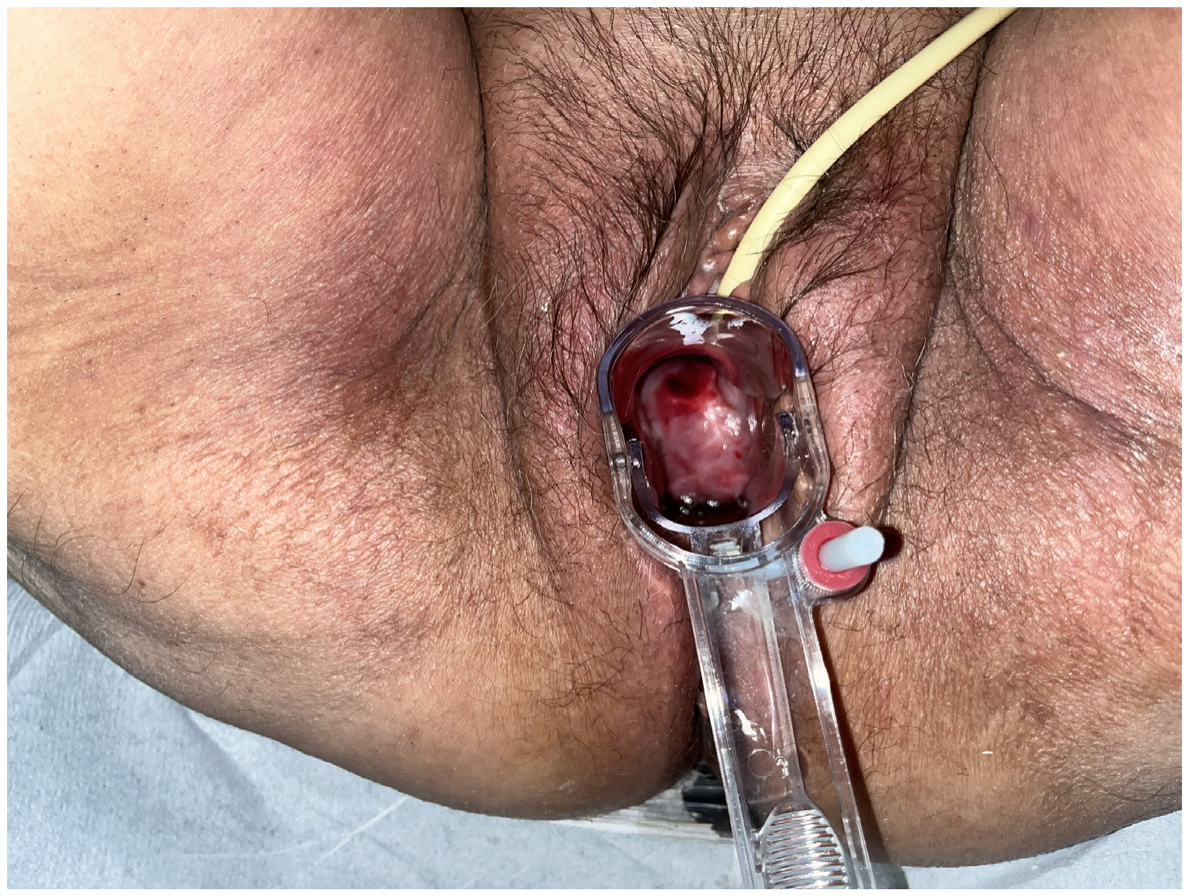
Pre-operative inspection of a large prolapsed pedunculated submucosal uterine leiomyoma: In the anatomical position of the cervix the presence of a large pelvic mass was observed, but the position of the external cervical os could not be precisely identified.

**Figure 2:**
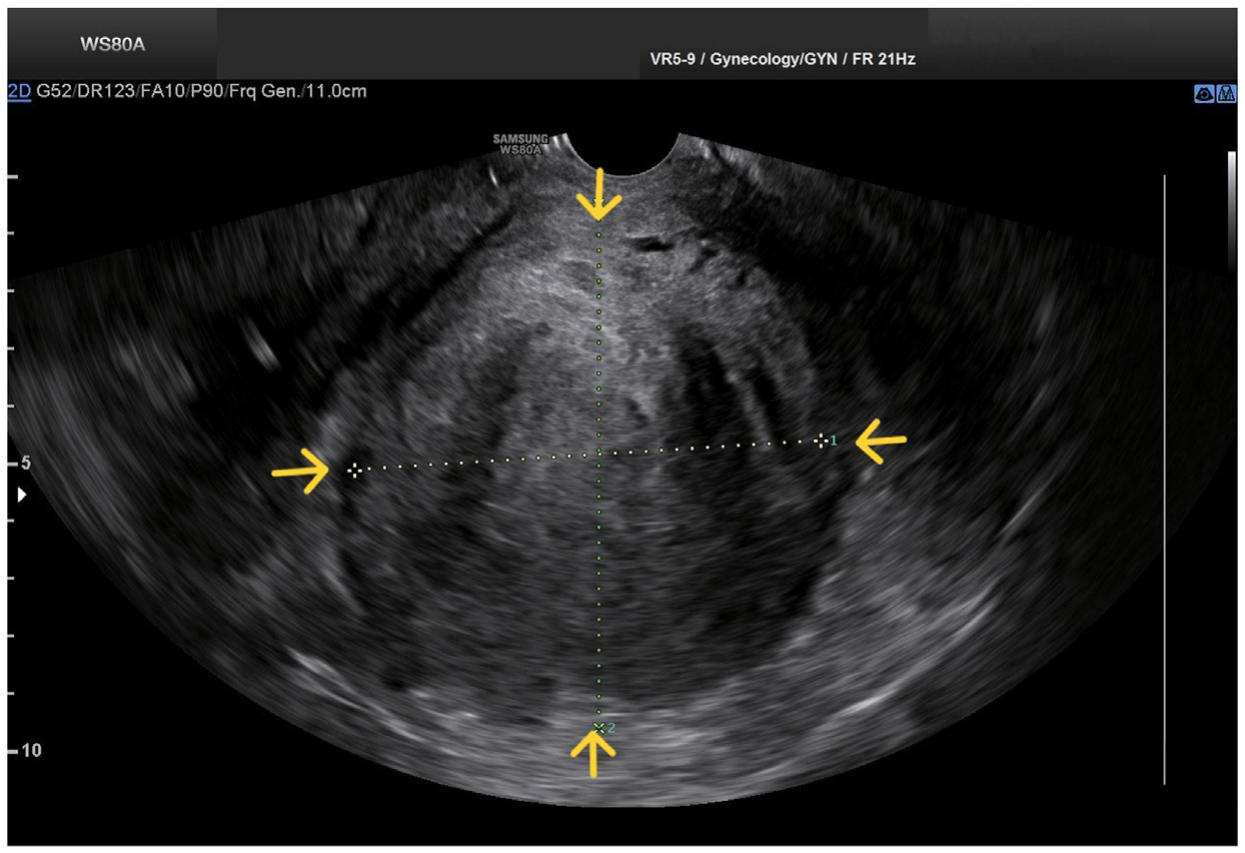
Transvaginal ultrasound image of a large prolapsed pedunculated submucosal uterine leiomyoma: The pelvic mass is clearly depicted (yellow arrows), without clear imaging of the adjacent anatomical structures.

**Figure 3:**
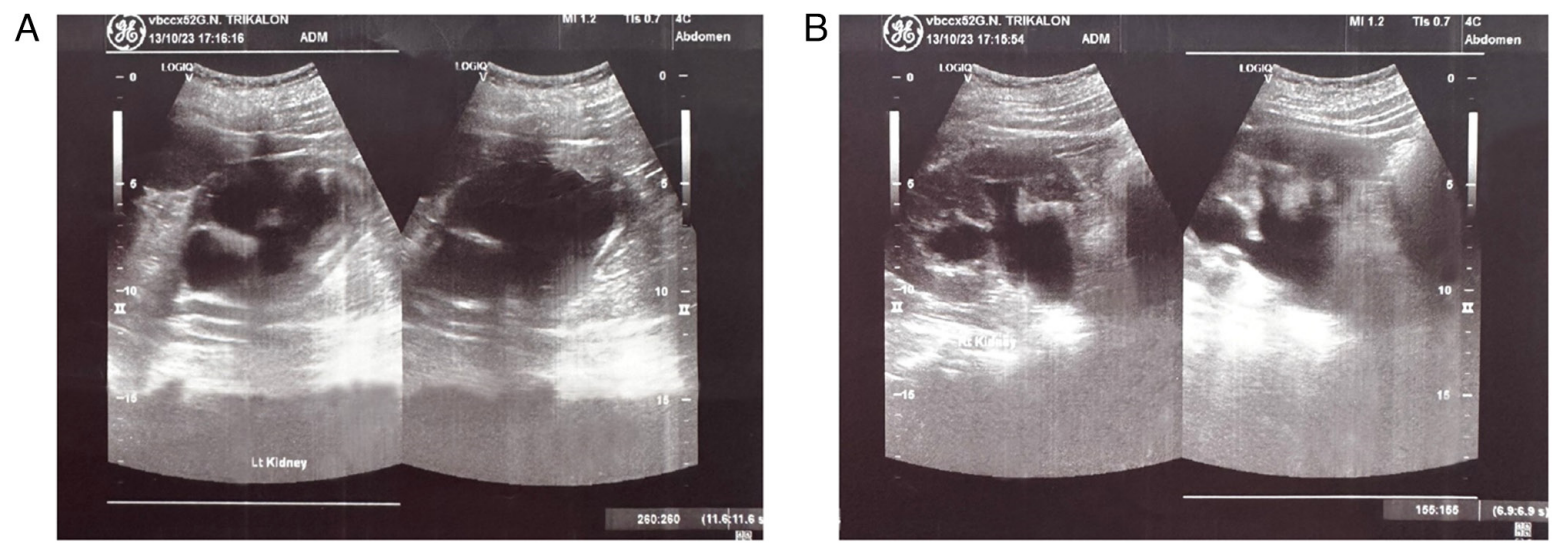
Preoperative ultrasound image of the kidneys revealing bilateral dilatation of the pelvicalyceal system and the upper ureters: (A) Right kidney, (B) left kidney.

**Figure 4:**
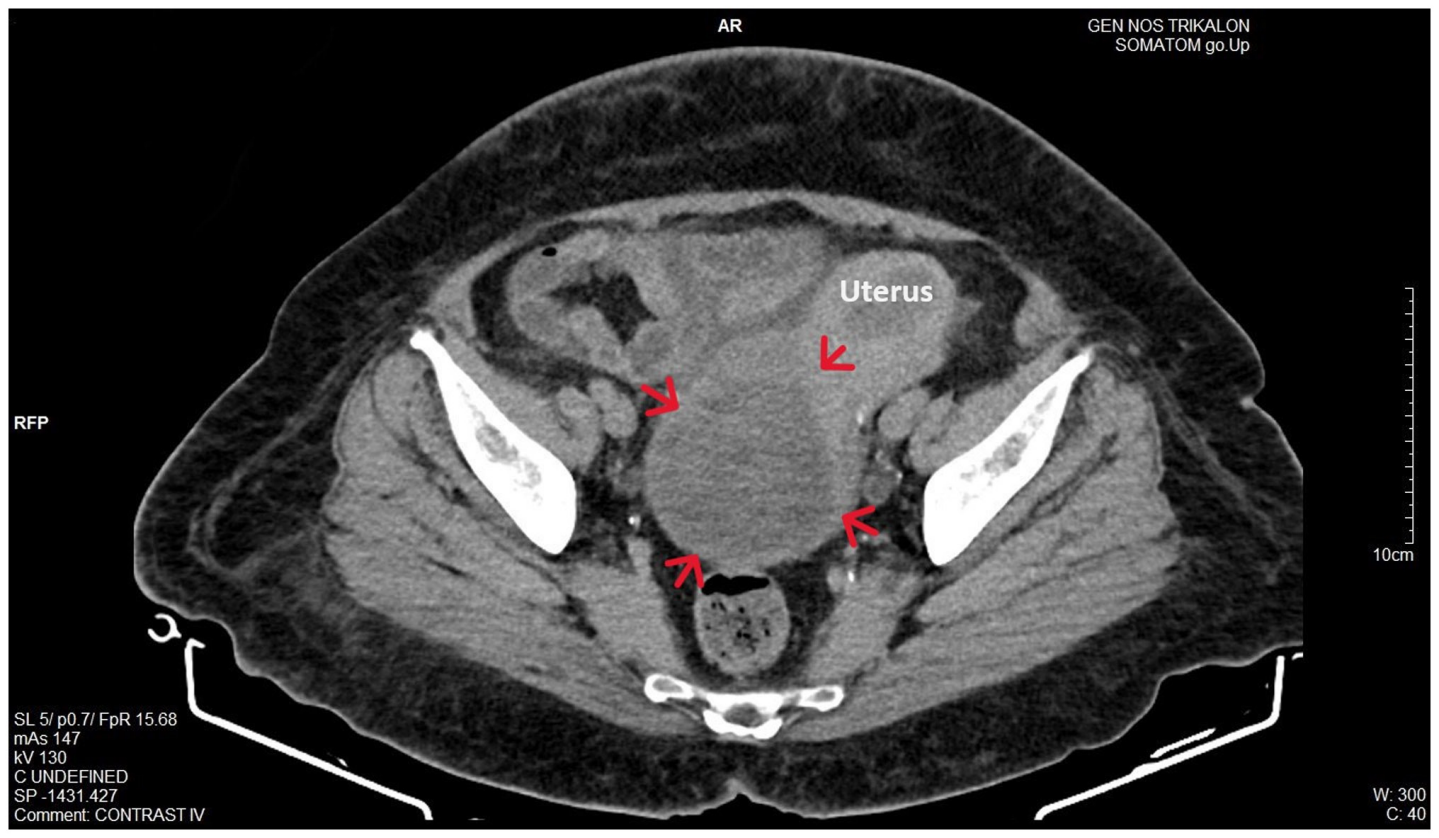
Computed tomography imaging of a large prolapsed pedunculated submucosal uterine leiomyoma: Significant distention and non-homogeneity of the cervix (red arrows) with thinning of the external wall is depicted.

**Figure 5:**
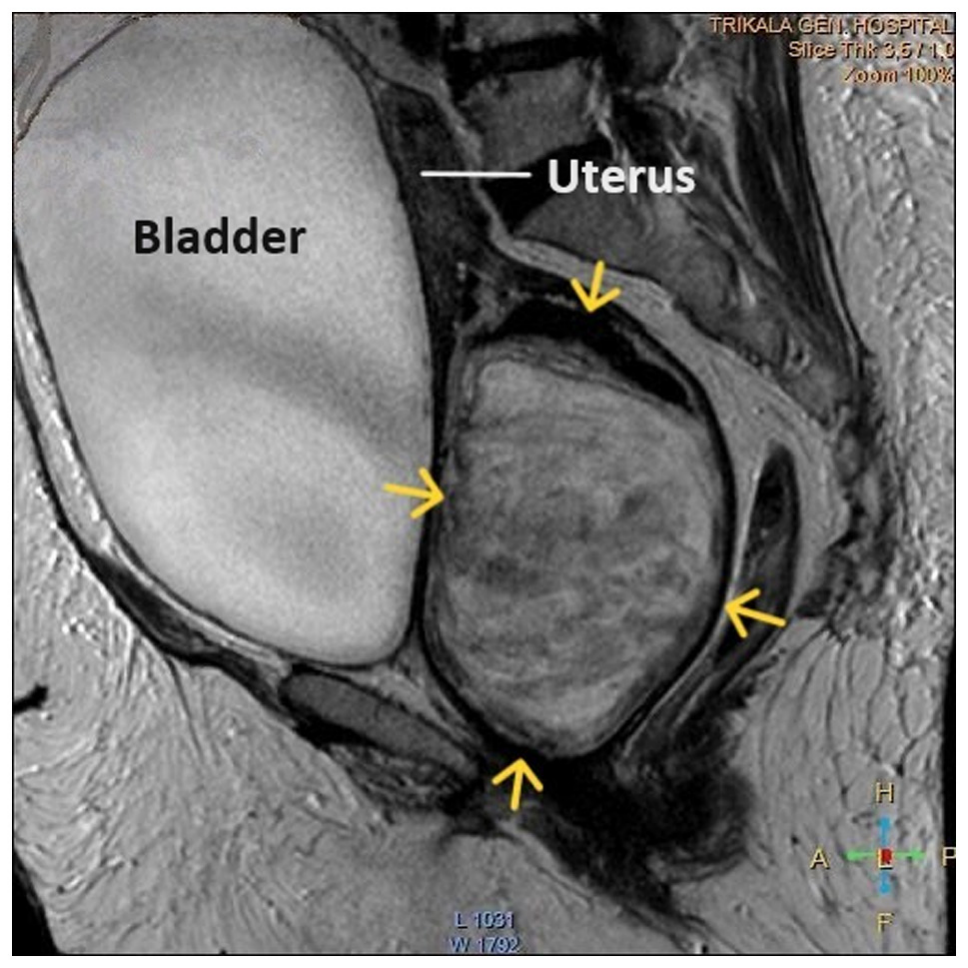
Magnetic resonance imaging of a large prolapse pedunculated submucosal uterine leiomyoma: The depicted urinary bladder distention with internal non-homogeneity of the cervical canal and presence of a lobulated lesion in the endocervix (yellow arrows) was misdiagnosed as an intracervical leiomyoma.

Upon a gynecological examination and upon the inspection of the vagina with a speculum, the cervix was not visible. In the upper third of the vagina, in the anatomical position of the cervix, a large solid mass was observed; the position of the external cervical os could not be clearly identified by visual inspection or palpation (Fig. 1). The transvaginal ultrasonographic findings were inconclusive. The scan detected the presence of a large well-circumscribed mass, with a maximum diameter of 10 cm, at the anatomical position of the cervix, raising suspicion of an intracervical leiomyoma (Fig. 2). A renal ultrasound revealed the bilateral dilatation of the pelvicalyceal system and the ipsilateral proximal ureter (Fig. 3). Furthermore, a computed tomography scan was performed, which revealed significant bladder dilatation and internal non-homogeneity of the cervical canal throughout its entire length, measuring 105x95x90 mm. This lesion caused the thinning of the external wall of the cervix and anterior displacement of the bladder. At the same time, it exerted compression on the posterior wall of the bladder, and malignancy arising from the cervix could not be excluded (Fig. 4). Additionally, computed tomography confirmed the dilatation of the pelvicalyceal system bilaterally up to the ureterovesical junction, accompanied by localized renal cortical thinning and lobulated contour of the left kidney. Magnetic resonance imaging was performed to further elucidate the findings of computed tomography. Magnetic resonance imaging revealed marked bladder distention with internal non-homogeneity of the cervical canal and the presence of a lobulated lesion that protruded intracanal with dimensions of 100x85x105 mm (Fig. 5). This finding was attributed to a large intracervical leiomyoma, strongly ruling out the possibility of cervical malignancy. The levels of tumor markers, namely carcinoembryonic antigen, cancer antigen 125, cancer antigen 15-3 and cancer antigen 19-9 were within the normal range.

**Figure 6:**
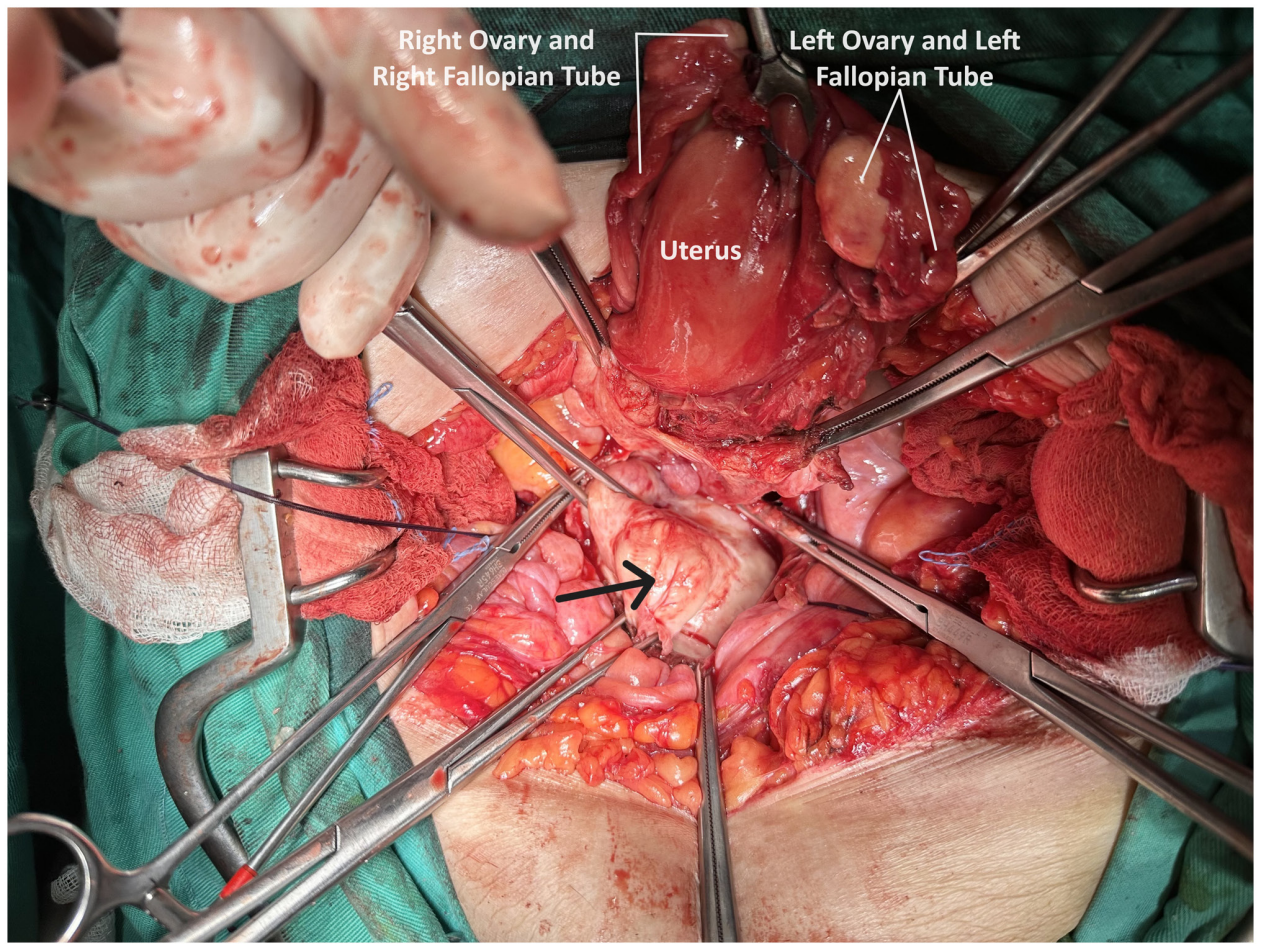
Intraoperative image of large prolapsed pedunculated submucosal uterine leiomyoma: Normal uterus and adnexa were observed, and the presence of a large pedunculated submucosal leiomyoma prolapsing into the vagina was identified (arrow).

The initial conservative management of uterine bleeding involved the administration of tranexamic acid (Transamin at 500 mg/5 ml injectable solution by Help Pharmaceuticals), two ampules in 0.9% NaCl solution infused intravenously three times a day for 4 days, the treatment of anemia through transfusion with 4 units of packed red cells and the use of broad-spectrum antibiotics, specifically cefoxitin (Mefoxil at 2 g/vial injectable solution by Vianex Pharmaceutical Manufacturers) administered intravenously every 8 h until the day of surgery. Due to the deterioration of renal function following obstructive uropathy, a ureteral stent was placed in the left ureter and a nephrostomy was performed on the right kidney by the urologists. The placement of the right nephrostomy was decided due to the unsuccessful access of the ureteral stent to the right ureter. Following the urological intervention, there was an improvement in renal function. The variations of urea and creatinine values are detailed in Table I. Subsequently, following the improvement of renal function and patient counseling, it was decided to perform an exploratory laparotomy. Intraoperatively, normal uterus and adnexa were observed, and the presence of a large pedunculated submucosal leiomyoma prolapsing into the vagina was identified (Fig. 6). Following the resection of the leiomyoma, an abdominal total hysterectomy with bilateral salpingectomy-oophorectomy was performed. At the end of the surgery, it was possible to place a ureteral stent in the right ureter as well.

**Figure 7:**
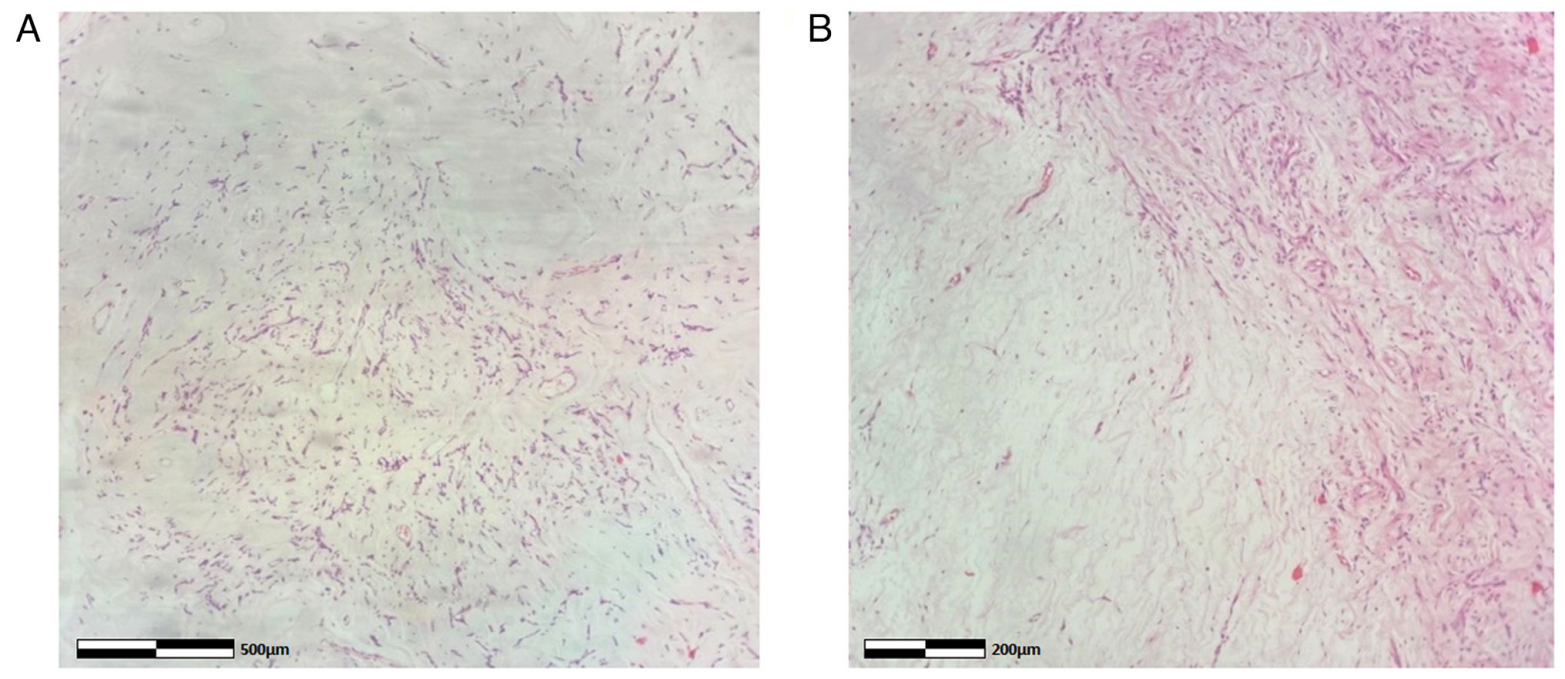
Histological image of a large prolapsed pedunculated submucosal uterine leiomyoma: Myxoid leiomyoma, hematoxylin and eosin staining. (A) magnification, x10; (B) magnification, x100 (different area of the tissue section).

The collected specimens were sent for histopathological analysis. This was carried out at the Anatomic Pathology Laboratory of the General Hospital of Trikala. The thickness of the obtained tissue sections was 5 µm and they were paraffin-embedded. A buffered formalin 10% solution was used as the fixative agent, for 36 h at room temperature. Hematoxylin and eosin 0.5% alcohol (Diachel A.E.) staining was used, at room temperature with a 12-min duration. All microscopic examinations were performed using a LEICA DM2000 optical microscope (Leica Microsystems GmbH). The histological examination of the surgical specimen confirmed the diagnosis of pedunculated submucosal uterine leiomyoma (Fig. 7). Supplementary immunohistochemical analysis was performed. The sections used were 4 µm in thickness, were paraffin-embedded and they were dewaxed for 40 min at 70˚C. Analysis was performed via the automated BOND-LEICA system (Leica Biosystems). Sections were placed sequentially in BOND™ Dewax solution, 100% v/v ethanol solution and BOND™ wash solution. For antigen retrieval, BOND™ Epitope Retrieval ER2 Solution, HIER, was used for cluster of differentiation 10 (CD10) and cytokeratin 7 (CK7) for 20 min in 100˚C, while ER1 solution (pH 7) for 20 min was used for smooth muscle actin (SMA) and estrogen receptor (ER). The block peroxide kit (Bond; Leica Biosystems) was used for 5 min. For CD10 antibody, protein block solution was used for 20 min. As regards primary antibody dilution this was: Dilution for CD10 (Menarini Hellas A.E.) ready-to-use antibody (cat. no. 44 217 CD10 RTU), for SMA (Zytomed Systems) ready-to-use (cat. no. 1A4 A00002-IFU-IVD-0002), for ER (Dako) 1:40 (cat. no. M3643) and for CK7 (Dako) 1:100 with proprietary Leica (Bond; Leica Biosystems) dilution agent. A duration of incubation of 30 min was used for all antibodies. The post-primary kit was used for a duration of 10 min at an incubation temperature of 100˚C. Subsequently, a secondary detection kit polymer (Bond; Leica Biosystems) was used for a duration of 10 min and the DAB kit (Bond; Leica Biosystems) for 10 min to facilitate visualization. Hematoxylin was applied for 5 min as a counterstain at room temperature and the sections were dehydrated, mounted and coverslipped. The resulting slides were examined under a LEICA DM2000 optical microscope (Leica Microsystems GmbH), at a magnification of x40, x100 and x400. The specimen demonstrated strong diffuse positive staining for SMA and ER, positive for CD10 and negative for cytokeratin 7 (CK7) (images not available; data not shown).

**Figure 8:**
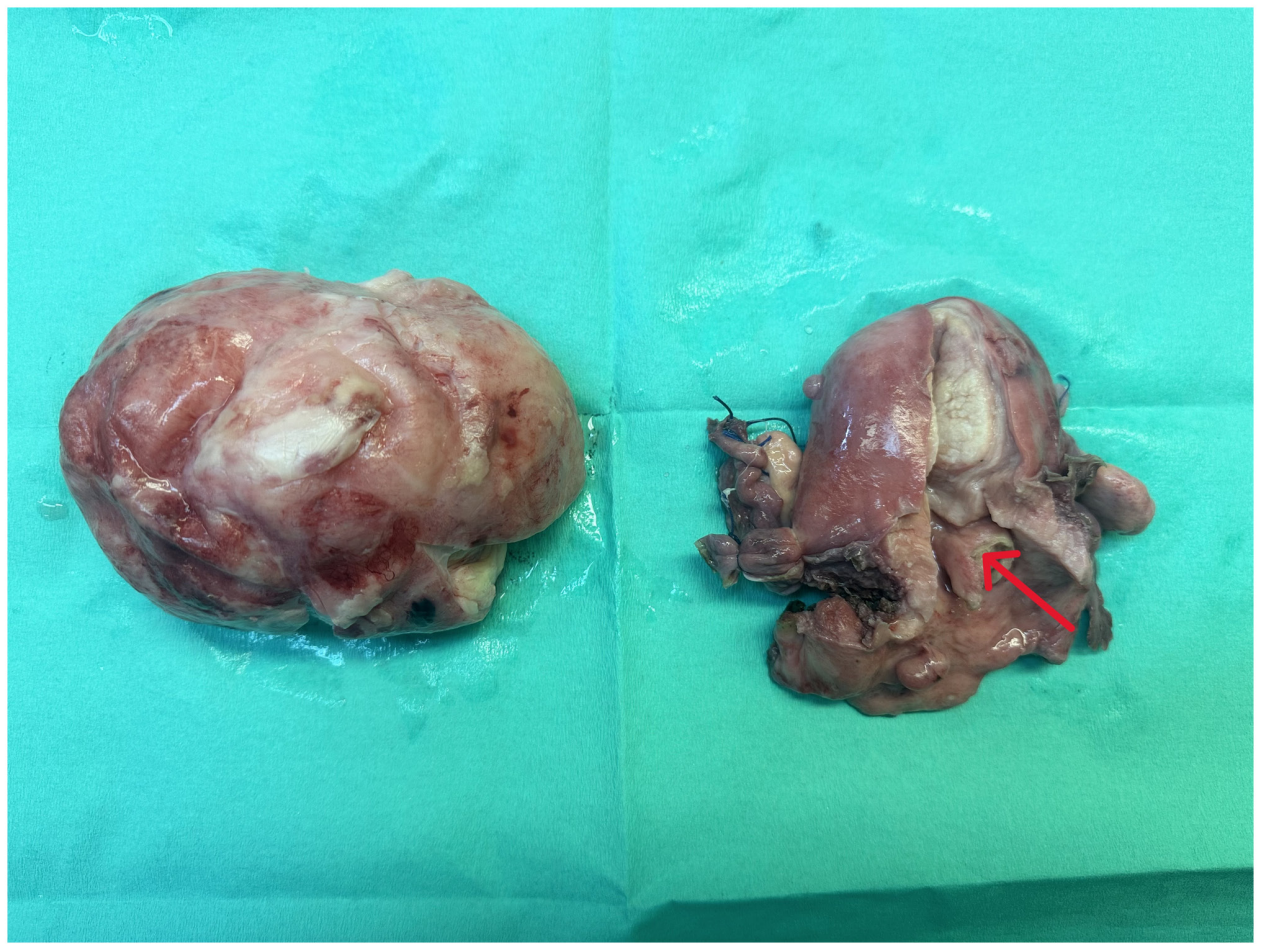
Surgical specimen of large prolapsed pedunculated submucosal uterine leiomyoma after an abdominal total hysterectomy with bilateral salpingectomy-oophorectomy: The presence of the pedicle (red arrow) is evident at the site of the uterine isthmus.

**Figure 9:**
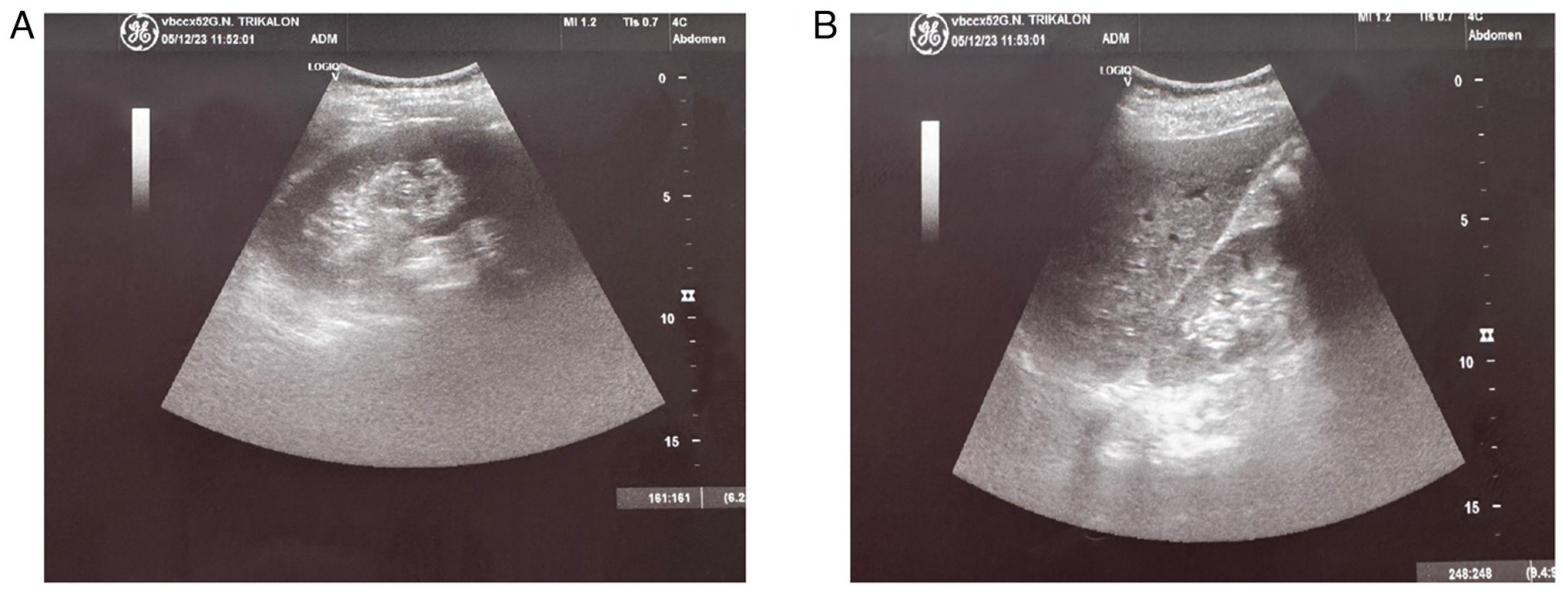
Post-operative ultrasound image of the kidneys, which shows complete recovery of renal morphology and function: (A) Left kidney, (B) right kidney.

Finally, the Alcian blue histochemical stain was used. Sections of 4 µm in thickness were dewaxed for 40 min at a temperature of 70˚C. The sections were placed in xylene for 10 min in room temperature. Subsequently, they were hydrated in decreasingly graded alcohols, submerged 10-15 times in each of the following solutions: 100% v/v ethanol, 96% v/v ethanol, 80% v/v ethanol and 70% v/v ethanol. Subsequently, the sections were washed using tap water and then distilled water, finally being ready for staining. A few drops of Alcian Blue stain [1% in 3% acetic acid (pH 2.5); Atom Scientific] were added and left there for 30 min. The sections were blotted and oxidized with 1% periodic acid solution for 10 min and then washed, firstly for 5 min using tap water and for 2 min using distilled water. Subsequently, Schiff reagent solution was added for 20 min; the section was washed with tap water for 5 min and rinsed with distilled water. Finally, Mayer's hematoxylin (Atom Scientific) was added for 5 min, and the section was washed in tap water for 5 min and rinsed in distilled water and finally, it underwent hydration in increasingly graded alcohols (same as before, in reverse), was mounted and coverslipped. The slides were studied under the same optical microscope (LEICA DM2000; magnification, x40, x100 and x400) as aforementioned. Alcian blue staining of the specimen ultimately revealed myxoid differentiation (images not available; data not shown). The immunohistochemical analysis, along with histological findings indicating no nuclear atypia, necrosis, mitoses, or invasive pattern, strongly suggested a diagnosis of leiomyoma with myxoid degeneration. These findings also supported the exclusion of other tumors, such as leiomyosarcoma. Macroscopically, an oval-shaped tumor with a maximum diameter of 10 cm exhibited a smooth outer surface entirely covered by serosa and the presence of a pedicle originating from the uterine isthmus was observed (Fig. 8). Following a smooth post-operative course and a distinct immediate improvement in renal function (Table I), the patient was discharged from the clinic on the 5th post-operative day. After 6 months, without the presence of ureteral stents, renal morphology and function had fully recovered (Fig. 9). The serum creatinine level was 1.1 mg/dl. The patient remains under regular follow-up evaluation at the Nephrology and Gynecology outpatient Department of the General Hospital of Trikala.

## Discussion

Uterine fibroids are the most common benign neoplasms of the uterus and a significant cause of morbidity among women of reproductive age, affecting up to 68.6% of the female population (4,5). Submucosal leiomyomas represent ~15-20% of these cases (6). Prolapsed uterine leiomyomas are pedunculated submucosal fibroids, which, depending on the length of the vascular pedicle, can descend through the cervical canal into the vagina or protrude outside the vaginal opening (7). The exact percentage of pedunculated submucosal leiomyomas that prolapse into the vagina via the cervix is not precisely known. A previous study reported that the estimated prevalence of prolapsed pedunculated submucosal uterine leiomyomas was 2.5% among patients undergoing surgery (8).

The clinical diagnosis of a prolapsed pedunculated submucosal uterine leiomyoma within the vagina can be challenging, particularly in cases where the leiomyoma is of A notable size and has a short vascular pedicle. Prolapse of a pedunculated submucosal leiomyoma through the cervix may either be asymptomatic or manifest with symptoms, such as vaginal bleeding, vaginal discharge, or pelvic pain (9). In the patient described in the present study, the association of a prolapsed pedunculated submucosal uterine leiomyoma with bilateral chronic obstructive uropathy and renal dysfunction appears to be unique in the English literature. Most likely, the large pedunculated submucosal fibroid that prolapsed into the vagina, at the site of the contiguity of the ureters with the upper segment of the vagina, caused severe and chronic ureteral compression, resulting in bilateral hydroureteronephrosis and renal dysfunction. In addition, in the patient described herein, the diagnosis of a prolapsed fibroid was challenging during gynecological examination. Initially, it was incorrectly hypothesized that the cervix was not visible due to its deformation by the large intracervical leiomyoma and the compression exerted by the vaginal walls. In fact, however, the solid mass observed upon the examination of the vagina with a speculum (Fig. 1), was the prolapsed leiomyoma and not the cervical wall thinned by the compression of the tumor. Due to its large dimensions and a short vascular pedicle, the prolapsed leiomyoma covered the entire upper third of the vagina, hindering both visualization and palpation of the cervix.

Consequently, it is considered that the diagnostic analysis of patients with prolapsed pedunculated submucosal uterine leiomyoma can be confusing, since the gynecological examination may not be able to exclude uterine prolapse or uterine inversion (7,10). Additionally, the elongation and torsion of the vascular pedicle may lead to hemorrhagic and gangrenous degeneration of the prolapsed submucosal leiomyoma, posing a significant challenge in the differential diagnosis from uterine leiomyosarcoma. Endometrial stromal tumor or giant endometrial polyps should often be included in the differential diagnosis of prolapsed pedunculated submucosal uterine leiomyoma (11). Furthermore, bilateral obstructive uropathy with renal dysfunction could more readily be attributed to a large intracervical uterine leiomyoma than to a prolapsed pedunculated submucosal leiomyoma in the upper third of the vagina (12).

Imaging plays a crucial role in the pre-operative diagnosis of patients with prolapsed pedunculated submucosal leiomyoma. In these patients, computed tomography can distinctly reveal a well-circumscribed pelvic mass with mixed echogenicity, incorporating degenerative changes and necrotic lesions, thereby facilitating an early and accurate diagnosis (13). Magnetic resonance imaging can also provide critical imaging information regarding the precise location of the leiomyoma in relation to the myometrium, the degree of vascularization and the position of adjacent anatomical structures, significantly contributing to the planning of optimal surgery (14,15). In the patient in the present study, however, the pre-operative imaging diagnosis of prolapsed pedunculated submucosal uterine leiomyoma posed significant challenges. None of the imaging modalities used (ultrasound, computed tomography or magnetic resonance imaging) was able to pre-operatively establish the diagnosis of prolapsed pedunculated submucosal uterine leiomyoma. The ultrasound initially misdiagnosed a large intracervical fibroid. Computed tomography confirmed the ultrasound findings, but could not exclude the possibility of cervical malignancy. Magnetic resonance imaging incorrectly confirmed the diagnosis of intracervical fibroid and almost ruled out the possibility of cervical malignancy.

The treatment of a prolapsed pedunculated submucosal leiomyoma of the uterus is typically surgical (myomectomy or abdominal total hysterectomy with bilateral salpingectomy-oophorectomy). Factors, such as the desire for fertility preservation, the absence of co-existing uterine leiomyomas and the small size of the prolapsed submucosal leiomyoma may favor the therapeutic option of vaginal myomectomy over abdominal total hysterectomy with bilateral salpingectomy-oophorectomy (16). The absolute indication for abdominal total hysterectomy is severe uterine bleeding, sepsis or an unsuccessful vaginal approach (17). Vaginal myomectomy is considered a safe and easy surgery for the treatment of prolapsed pedunculated submucosal uterine leiomyoma, as it is associated with a low recurrence rate and minimal morbidity (18,19). Conversely, the increased risk of intraoperative hemorrhage that may be associated with some pedunculated submucosal leiomyomas can be controlled with hysteroscopy. Vaginal myomectomy followed by an operative hysteroscopy to complete the resection of the leiomyoma's residual vascular pedicle, following gentle cervical torsion around the hysteroscope, which prevents the leakage of the distension liquid, provides excellent visibility for hemorrhage control and ensures the complete resection of the pedicle. This approach significantly reduces the risk of recurrence (20).

In the patient described herein, the scarcity of sufficient published data regarding the association between prolapsed submucosal uterine leiomyoma and severe obstructive uropathy with renal dysfunction was the main reason for both the diagnostic and therapeutic challenges that were encountered. Consequently, due to the pre-operative misdiagnosis of a pedunculated submucosal uterine leiomyoma as an intracervical fibroid, performing an exploratory laparotomy emerged as the only treatment option for the patient. There was no option between abdominal total hysterectomy with bilateral salpingo-oophorectomy and vaginal myomectomy. The diagnosis of a prolapsed pedunculated submucosal uterine leiomyoma in the patient described herein was made intraoperatively, following the dissection of the anterior vaginal wall and access into the vagina (Fig. 6). Nevertheless, it is noted that the presence of a short and thick vascular pedicle originating from the uterine isthmus, combined with the presence of a large leiomyoma are conditions that may not ensure the success of a myomectomy via vaginal approach.

There are certain possible limitations to the present study that should be mentioned. The present study reports a very rare case of a large pedunculated submucosal uterine leiomyoma with prolapse into the vagina, which was associated with bilateral obstructive uropathy and severe renal dysfunction. The extremely rare manifestation of bilateral obstructive uropathy accompanied by severe renal dysfunction that can be caused by prolapse of a large pedunculated submucosal leiomyoma into the vagina created a serious differential diagnostic challenge compared with intracervical leiomyomas and led to misdiagnosis. It is considered that the association of a prolapse of pedunculated submucosal uterine leiomyoma with chronic bilateral obstructive uropathy and renal dysfunction appears to be a unique clinical case in the English literature. In addition, during the gynecological examination, the prolapsed leiomyoma due to its large size and short vascular pedicle did not allow either the inspection or palpation of the cervix. Furthermore, the bilateral obstructive uropathy with renal dysfunction that presented in the patient could more easily be attributed to a large intracervical uterine leiomyoma than to a prolapse of a pedunculated submucosal leiomyoma into the vagina. Additionally, in the patient described herein, none of the imaging modalities used were able to prevent a misdiagnosis of intracervical leiomyoma preoperatively. It is likely that the lack of sufficient published data regarding the association between the prolapse of pedunculated submucosal uterine leiomyoma and obstructive uropathy with severe renal dysfunction was the main reason for the diagnostic difficulties that were encountered. Therefore, although uterine submucosal leiomyomas prolapsing into the vagina are common, in the patient described herein, the diagnosis was made at a late stage and intraoperatively.

In conclusion, the case presented herein, involving bilateral obstructive uropathy with renal dysfunction associated with large prolapsed pedunculated submucosal leiomyoma of the uterus, is unique in the English literature, at least to the best of our knowledge. A pre-operative diagnosis, particularly in cases where the leiomyomas are large and prolapse into the vagina, poses several challenges. A thorough clinical and imaging preoperative evaluation is crucial for the early diagnosis of hydroureteronephrosis and renal dysfunction attributed to a pelvic tumor. Early and accurate diagnosis facilitates the immediate utilization of the optimal treatment options, preventing permanent renal damage and ensuring the continued health and quality of life of these patients.

## Figures and Tables

**Table I:** Pre- and post-operative laboratory test results of the patient with prolapsed pedunculated submucosal leiomyoma of the uterus.

Laboratory tests	Day of admission to the clinic	3rd day of hospitalization	Post-operatively	2nd Post-operative day after laparotomy	5th post-operative day after laparotomy	3 Months after surgery	6 Months after surgery	Normal laboratory values
Ht	15.4%	29%	32.5%	30.1%	28.7%	31.2%	33.6%	37.7-49.7%
Hb	4.1 g/dl	9.6 g/dl	10.1 g/dl	9.5 g/dl	8.7 g/dl	10.5 g/dl	11.1 g/dl	11.8-17.8 gr/dl
PLT	483x10^3^/ml	327x10^3^/ml	400x10^3^/ml	357x10^3^/ml	382x10^3^/ml	257x10^3^/ml	241x10^3^/ml	150-350x10^3^/ml
WBC	17.3x10^3^/ml	11.9x10^3^/ml	9.7x10^3^/ml	11.3x10^3^/ml	7.4x10^3^/ml	8.9x10^3^/ml	8.5x10^3^/ml	4-10.8x10^3^/ml
NEUT	85.9%	88.3%	61%	90.4%	65.8%	56.7%	54%	40-75%
CRP	0.67 mg/dl	0.51 mg/dl						<0.7 mg/dl
APTT	34.8 sec	34.1 sec	27.8 sec				29.3 sec	24.0-35.0 sec
INR	1.25	1.18	1.00				1.01	0.8-1.2
FIB	316 mg/dl	351 mg/dl	498 mg/dl				358 mg/dl	200-400 mg/dl
U	64 mg/dl	71 mg/dl	61 mg/dl	55 mg/dl	41 mg/dl	40 mg/dl	37 mg/dl	10-50 mg/dl
Cr	1.74 mg/dl	2.87 mg/dl	1.95 mg/dl	1.63 mg/dl	1.28 mg/dl	1.21 mg/dl	1.1 mg/dl	0.40-1.10 mg/dl
Κ^+^	3.7 mmol/l	4.1 mmol/l	4.8 mmol/l	5.1 mmol/l	4.5 mmol/l	3.9 mmol/l	3.7 mmol/l	3.5-5.1 mmol/l
Να^+^	138 mmol/L	141 mmol/l	138.4 mmol/l	134.4 mmol/l	138.6 mmol/l	138 mmol/l	139.2 mmol/L	136-145 mmol/l
B	0.45 mg/dl	0.85 mg/dl	0.42 mg/dl				0.38 mg/dl	0.3-1.2 mg/dl
AST	32 IU/l	37 IU/l	29 IU/l				28 IU/l	5-33 IU/l
0ALT	35 IU/l	41 IU/l	10 IU/l				25 IU/l	10-37 IU/l

The post-operative improvement of the patient's renal function is evident. Ht, hematocrit; Hb, hemoglobin; PLT, platelets; WBC, white blood cells; NEUT, neutral; CRP, C reactive protein; APTT, activated partial thromboplastin time; INR, international normalized ratio; FIB, fibrinogen; U, urea; Cr, creatinine; K^+^, potassium; Na^+^, sodium; B, bilirubin; AST, aspartate transaminase; ALT, alanine aminotransferase.

## Data Availability

The datasets used and/or analyzed during the current study are available from the corresponding author on reasonable request.
